# The Effect of Grazing Intensity and Sward Heterogeneity on the Movement Behavior of Suckler Cows on Semi-natural Grassland

**DOI:** 10.3389/fvets.2021.639096

**Published:** 2021-03-26

**Authors:** Dina Hamidi, Martin Komainda, Bettina Tonn, Jens Harbers, Natascha Alexandria Grinnell, Johannes Isselstein

**Affiliations:** ^1^Department of Crop Sciences, Grassland Science, University of Goettingen, Göttingen, Germany; ^2^Center of Biodiversity and Sustainable Land Use, University of Goettingen, Göttingen, Germany

**Keywords:** herbage allowance, GPS tracking, precision livestock farming, walking distance, spatial distribution

## Abstract

Extensively grazed semi-natural grasslands contribute to a wide range of ecosystem services, including the preservation of biodiversity and provision of livestock feed. Depending on the grazing intensity, cattle are set in motion to fulfill their nutritional needs. In this way, they influence the vegetation composition, while at the same time the foraging behavior is affected by the vegetation. A better understanding of the relationship between grazing intensity and animal behavior is an essential component for strategies to improve the value of semi-natural grasslands and for gaining insights for the development of smart farming technologies. The long-term cattle grazing experiment “FORBIOBEN” with its replicated three paddock-scale (1 ha) grazing intensities [moderate (M), lenient (L), very lenient (VL)] was used to investigate the movement behavior of suckler cows during four grazing periods between 2017 and 2020. For this, pregnant suckler cows (Fleckvieh) were equipped with Vectronics GPS Plus (VECTRONIC Aerospace GmbH, Berlin) collars, which recorded the position of the animals at defined time intervals. The main outcomes were that with an increase in the grazing intensity, the herbage on offer declined and, consequently the herbage allowance. However, the spatial heterogeneity of the herbage on offer decreased with increasing grazing intensity (M < VL) which means that the amount of available herbage was lower but more evenly distributed under moderate grazing. Further, there was a tendency that the moderate grazing intensity was associated with the highest effort of walking compared to lenient and very lenient grazing in three out of four grazing periods. We found a strong (*p* < 0.001) negative correlation among walking distance vs. herbage variability across all treatments × periods. Consequently, the grazing intensity itself was not a good predictor of walking distances which were mainly a result of the available herbage, its distribution or heterogeneity. Future smart farming livestock management systems will, therefore, likely require interfaces with the grassland growth rates and heterogeneity benchmarks if decisions based on livestock movement should be reliable.

## Introduction

Grassland is the largest terrestrial biome, covering ~3.2 billion ha worldwide (1) and a large part of this area is used by grazing herbivores. Depending on the environmental conditions, the animal species and grazing method, these grazing herbivores influence the sward while their performance, on the other hand, is influenced by the sward properties (2, 3). Extensification of grassland leads to a shift towards a more diverse botanical composition and increased plant species richness (4). For instance, extensively grazed semi-natural grasslands host a great number of plant species, which is why they essentially contribute to the biodiversity of agricultural landscapes (5, 6). The vegetation often develops into a heterogenous pattern of different sward height classes of tall and short patches (7) which results from the so-called patch grazing (8). Patch grazing is characterized by a pronounced spatial heterogeneity in forage intake (9) with intensive and extensive grassland utilization occurring in close proximity within the same pasture. Several studies in semi-natural grassland found that the productivity (10), soil nutrient contents (10, 11), and the vegetation composition (4, 12) are driven by these temporally stable patches (7) rather than by the pasture-scale grazing intensity. The extent of patch grazing is controlled by the pasture stocking rate, i.e., the herbage allowance per grazing animal. It has been shown that under low stocking rates, animals tended to graze only on short grass patches even at the end of the grazing season (13). This indicates that the cattle regularly return to the same spots of high-quality herbage. Assuming that the productivity of these patches is maintained, the effort for foraging is low and the walking distances should mainly depend on the spatial distribution of these patches. With a more restrictive herbage allowance, i.e., higher grazing pressure, the animal has to visit more places every day to fulfill its energy demand because less herbage is available per patch so that more movement is required. On the other hand, a higher herbage allowance per animal does not always result in less movement since in a patchy grassland the foraging areas are spatially distributed (7).

Hejcmanová et al. (14) investigated behavioral patterns under extensive and intensive continuous grazing (fewer vs. more cattle per pasture) and found a clear trend towards longer grazing time under intensive grazing. However, in a study of Dumont et al. (13), the walking distances per grazing event were not affected by the stocking rate and group size. Thus, it remains an open question to what extent the grazing intensity and, hence, the availability or distribution of herbage control the activity of grazing cattle in semi-natural grassland ecosystems. Such information is needed if any decision support tools in future smart farming systems will be based on the spatial animal movement.

Using GPS (Global Positioning System) collars to track the spatial behavior of grazing cattle is a well-established method to investigate the drivers of animal behavior. Since 1978 GPS is operational and since 1984 civilian use is allowed. The University of Kentucky began to use GPS collars for cattle tracking in the 1990s to be able to integrate spatial information into cattle management procedures (15). Using GPS collars in studies of animal movement has many benefits: individuals can be tracked over a long-term period with predefined time intervals and automatically recorded geographical positions (16), which is very helpful information on large pastures and rangelands (17). In addition, accurate and efficient information on grazing behavior can be provided by the use of GPS for monitoring of grazing animals (18). Animal-related GPS recordings in combination with a geographic information system (GIS) can provide information on spatial interrelations of animal behavior and the vegetation (19). In recent years, several studies have investigated the potential of GPS tracking data to deduce behavioral patterns of grazing cattle. Homburger et al. (20, 21), both based on investigations in heterogeneous subalpine pastures, recommended to differentiate only grazing and resting when using GPS tracking. Walking is mainly correlated with grazing because cattle always walk several steps between bites while walking without grazing is a relatively rare activity (22). In the study by Homburger et al. (20), only 6.7% of movement was accounted for by walking without grazing as assessed by visual observations. In another study (17) it was shown that the time budgets of the main cattle behavior (grazing, resting, walking) were not influenced by the grazing management. However, the walking distances were affected in that study and also in that by Baudracco et al. (23), where cows on a pasture with lower herbage allowance spent more time walking. Consequently, assessing movement patterns in terms of walking distances will provide a reliable indicator for the effort of the grazing cattle to fulfill dietetic demands under conditions of varying herbage allowances. Moreover, such assessments can help to identify the driving forces of livestock movement, including the role of sward characteristics. The study presented here was conducted in a multi-year grazing experiment with livestock cattle on semi-natural grasslands under three different grazing intensities, defined by different target sward heights (moderate: 6 cm, lenient: 12 cm, very lenient: 18 cm) resulting in decreasing stocking rates (moderate to very lenient). The grazing experiment was established in 2002 under the EU framework 5 research project “FORBIOBEN” (3). The aim of “FORBIOBEN” with its three paddock scale grazing intensities is to represent the entire gradient of grassland extensification. Over three seasons (2017, 2019, 2020), cattle were equipped with GPS collars with the aim to disentangle interactions between the grazing intensity and cattle movement by taking into account both herbage allowance and the spatial variability of the herbage on offer. We hypothesized that (i) cattle activity increased with lower herbage allowance because the area, size and stability of tall patches increase with decreasing grazing intensity (7), and foraging resources are the most obvious drivers of grazer distribution at pasture (8), we further hypothesized that (ii) the spatial distribution of cattle during activity (grazing) peaks is more even under moderate compared to lenient grazing intensity.

## Materials and Methods

### Experimental Site, Setup and Weather Conditions

The present study investigated the movement behavior of suckler cows in response to three different grazing intensities. It was carried out over four periods between spring 2017 and spring 2020 as part of the grassland experiment “FORBIOBEN,” which is located at the experimental farm of the University of Göttingen in Relliehausen, Solling Uplands, Lower Saxony, Germany (51°46'55.9 “N, 9°42'11.9”E), 250 m above sea level. The vegetation is a moderately species-rich semi-natural grassland classified as *Lolio-Cynosuretum*. The three most important grasses in 2017 were *Festuca rubra, Lolium perenne* and *Cynosurus cristatus*, while the three most important dicot species were *Taraxacum officinale, Trifolium pratense*, and *Galium mollugo*. In 2020 this changed slightly towards *F. rubra, Dactylis glomerata* and *L. perenne* and for the dicots to *T. officinale, Lotus corniculatus*, and *Galium mollugo*.

The longtime climatic averages (yearly) of the German weather service ‘Deutscher Wetterdienst’ reference period (1991–2020), measured approximately 21 km apart, were: precipitation: 764 mm, temperature: 9.8 °C, sunshine hours: 1500 (24). Weather conditions in the investigated periods are summarized in Table 1. The grazing experiment “FORBIOBEN” was established in 2002 (3) and is maintained in its current state since 2005. It compares three intensities of cattle grazing described by different target vegetation heights, hereafter M: moderate grazing (6 cm), L: lenient grazing (12 cm) and VL: very lenient grazing (18 cm target vegetation height). The three grazing intensities are replicated in a randomized block design of three paddocks (1 ha each) per grazing intensity. The general framework of the “FORBIOBEN” experiment is extensive grassland management as no fertilizer, pesticide or any sward improvement measure is applied. Within this framework, the different grazing intensities represent the following strategies. Moderate grazing is aiming at reasonable agronomic performance; lenient grazing does not make full use of the herbage, leaving remaining herbage for biodiversity targets, and very lenient grazing is representing the minimum grazing intensity that is required to keep the grazing land open, i.e., maintain the open character of the grassland. The management is a continuous grazing system with a put-and-take approach. In this system, cattle are added to the paddocks when the target vegetation height is exceeded and removed when the vegetation height falls below the target.

**Table 1:** Weather conditions (TM: mean daily temperature (°C) and precipitation sum (mm) during the four investigated periods recorded by the meteorological station in Bevern 51°51′10.8”N 9°29′42.0”E coordinated by the German Weather Service 'Deutscher Wetterdienst' (DWD), 21 km from the experimental site.

**Period**	**TM (^**°**^C)**	**Radiation (W m^**2**^)**	**Precipitation sum (mm)**
2017	16.2	19,950.6	52.1
2019 spring	16.1	19,722.2	77.0
2019 autumn	12.6	13,004.0	16.0
2020	17.3	17,875.0	79.6

### Animals

During each stocking season (April/May – September/October), up to 27 pregnant, non-lactating Fleckvieh suckler cows grazed in all three grazing intensities. Usually, the target sward height of 6 cm in M is reached faster in spring, so that this treatment can be stocked earlier. The VL treatment was stocked when the target height of the L treatment was reached, to prevent natural succession of the grassland. Outside the grazing period, from November to April, the animals are in winter housing. Calving takes place in November and December; mating is in February and March. Cows return to pasture in mid-April, after weaning. Animals that were removed from the experimental paddocks because sward heights fell below the target values grazed an area adjacent to the experimental paddocks. During the investigated periods, the cows were randomly assigned to groups and distributed among the paddocks. Average stocking densities of the different grazing intensities during the investigation were, moderate grazing: 4.6 LU ha^−1^, lenient grazing: 3.8 LU ha^−1^, very lenient grazing: 2.7 LU ha^−1^ (LU: livestock unit, 500 kg live weight). A detailed overview is given in Table 2. The respective stocking rates under moderate, lenient and very lenient grazing, calculated as (LU × days on pasture) per year and pasture area, were 1.4, 0.5, and 0.4 LU ha^−1^a^−1^ in 2017; 0.9, 0.6 and 0.4 LU ha^−1^a^−1^ in 2019; and 0.7, 0.4 and 0.2 LU ha^−1^a^−1^ in 2020.

**Table 2:** Overview of the grazing management and treatments during the investigated periods and annual stocking rates.

**Period (duration)**	**GI**	**Age years ± sd**	**LW kg ± sd**	**SD (LU ha^**−1**^)**	**SR (LU ha^**−1**^ a^**−1**^)**
2017 (18.05–14.06)	M	4.8 ± 1.6	666.3 ± 73.3	5.3	1.4
	L	5.6 ± 2.8	638.7 ± 96.2	3.8	0.5
	VL	5.4 ± 1.2	658.3 ± 86.0	2.6	0.4
2019 spring (24.05–27.06)	M	6.0 ± 2.5	684.8 ± 97.0	5.5	0.9
	L	5.7 ± 2.4	667.0 ± 101.0	4.0	0.6
	VL	5.2 ± 2.2	638.0 ± 57.4	2.6	0.4
2019 autumn (06.09–22.09)	M	5.1 ± 2.7	749.3 ± 105.0	4.5	0.9
	L	6.2 ± 2.6	795.1 ± 60.8	4.8	0.6
	VL	5.5 ± 2.1	748.7 ± 91.2	3.0	0.4
2020 (11.06–12.07)	M	3.4 ± 1.3	620.0 ± 69.7	2.5	0.7
	L	6.0 ± 2.9	626.7 ± 80.6	2.5	0.4
	VL	7.8 ± 1.2	673.5 ± 44.6	2.7	0.2

*GI, grazing intensity; LW, live weight; SD, stocking density; LU, livestock unit; SR, stocking rates*.

### Collecting Data

The duration of the investigated periods differed in response to the weather conditions and, hence, the herbage growth (Table 2). Each period lasted for 28, 35, 17, and 32 days in 2017, 2019 spring, 2019 autumn and 2020, respectively. To avoid bias from acclimatization to the collars and increased movement associated with paddock changes, the data collected on the first and last day of each period were excluded. The dates shown in Table 2 omit these days and correspond to the actual daily data used.

At the beginning of each period, one cow per grazing intensity and replicate was equipped with a Vectronics GPS Plus (VECTRONIC Aerospace GmbH, Berlin) collar (weight: 1.36 kg), attached to the neck of a randomly chosen cow per paddock, corresponding to a total of nine GPS collars. In the periods 2019 spring and 2020 two collars, and in 2019 autumn one collar were found not to have recorded data when the collars were removed. The collars are equipped with internal devices for GPS localization and an activity sensor (three-way accelerometer). Every 128 s (2017 and 2019 spring), or every 60 s (2019 autumn and 2020), the GPS sensors in the collar recorded a signal about the location of the animal within the pasture. Each GPS data point was recorded with date, time, distance, speed, absolute and relative angle between two successive path segments. In addition, the activity sensor in the collar recorded data in 64-s intervals. For each interval, it measured the proportion of time that the head tilt angle of the animal exceeded 15°, i.e., the time that the head was not lowered. At the end of the respective grazing period, the collars were removed to retrieve data and analyzed to measure the activity in terms of walking distance.

Walking distance (m) per animal was measured at two temporal scales, per day and also per hour within day. Geographic coordinates were available in the Universal Transverse Mercator coordinate system (UTM) format. To calculate the distance between two sequential positions, the Pythagorean theorem was used. The results were summed for hourly and daily (24-h) periods.

Data obtained from the activity sensor of the collar in spring 2019 were used to assess the relationship between walking distance per hour and the duration of grazing in minutes per hour, following Homburger et al. (21). Measurement intervals during which the activity sensor reported a lowered head at least half of the time were classified as grazing. This classification was validated by visual observations during 2016.

### Sward Herbage Measurements and Sward Characteristics

To determine the grassland herbage on offer, a double sampling approach was conducted from early April to October. For this, the compressed sward height (CSH) was measured every 2 weeks using a rising plate meter of 30 cm diameter and 200 g plate weight (25) at 50 places randomly distributed in each paddock. Approximately every 4–8 weeks, the standing herbage dry matter was determined at six to eight random points per paddock. Biomass was cut manually at 1 cm above the soil surface in a 30-cm diameter ring after first measuring CSH at this location. This procedure was conducted in order to calibrate the relationship between CSH and grassland herbage mass based on linear regression models (26, 27). The herbage biomass samples were oven-dried at 60°C for 48 h to obtain the dry matter weight. Based on the relationship between CSH and standing herbage dry matter, the available herbage on offer (herbage mass) was modeled for every other date and CSH measurement without calibration sampling so that 50 herbage values were available per paddock on each date of CSH measurements. Herbage biomass prediction from CSH was reasonable (RMSE = 70.4 g m^−2^ and mean R^2^_adj_ = 0.63 averaged over all periods). The derived herbage on offer per CSH sampling point was used to calculate the spatial heterogeneity of the herbage on offer by calculating the standard deviation within paddock (SD herbage).

Botanical composition in ten 1-m^2^ quadrats was assessed in accordance with the method of Scimone et al. (28) with average proportions between 2017 and 2020 of 59.7 ± 9.6, 59.2 ± 13.5, and 53.7 ± 10.9% grasses and of dicotyledonous species of 26.1 ± 5.7, 27.8 ± 6.8 and 25.7 ± 6.2 (± SD) in M, L and VL, respectively. Further studies showed that within grazing intensities, the botanical composition differed between short and tall patches as a consequence of modified resource availability for light and soil nutrients (4). Tonn et al. (11) observed larger phytodiversity in short patches compared to tall ones, and Perotti et al. (29) found that species in tall patches had higher competitiveness and the ones in short patches higher stress tolerance according to the competitor, stress tolerator, ruderal (CSR) theory after Grime (30).

The *in vitro* organic matter digestibility as assessed using near-infrared reflectance spectroscopy in ten continuous observation plots of 1 m^2^ size per paddock were 78.5 ± 7.4, 76.2 ± 6.2 and 74.6 ± 6.0% (mean ± SD) on average over 2017 to 2020 in M, L and VL, respectively. No patch-specific forage quality data was assessed in the present study. We know, however, from the beginning of the grazing experiment, that tall and short patches differ in the stem-to-leaf ratio toward the end of the growing season (27) with consequences for forage quality (3). Pavlu et al. (31) indicated differences in patch-specific forage quality and a recent study by Ebeling et al. (10) on the same site 12 years after extensive grazing revealed that the short patches were less productive and likely remained in a vegetative state as a consequence of selective grazing.

### Data Analysis

Statistical analyses were carried out with the software R (32). Linear mixed effects models were calculated for each target variable using the package “nlme” (33). For this, every period was analyzed separately. Outliers were eliminated if present by considering values ranging 1.5-fold above the 75th or below the 25th percentile of the interquartile range (34). For all analyses, ~ <5% of the data were excluded as outliers. Normality of the residuals was checked by visual inspection of quantile–quantile plots. Variance homogeneity was evaluated by plots of residuals vs. fitted values and residuals vs. predictor values (35). Multiple contrast tests according to Tukey's test for significant influencing factor levels were followed using the “emmeans” package (36) after analysis of variances.

The daily distance was regressed on the fixed effects of grazing intensity and date as well as their interaction. The cow nested in block was modeled as a random effect in order to account for correlation between measurements on the same object. Then model reduction was performed from the global model using the MuMIn package (37). The model with the lowest AICc was chosen as the final model.

To assess the diurnal patterns within days, models with fixed effects of grazing intensity, hour per day and their interaction and the random effect of the block and cow nested in block were generated. The dates per period were treated as replicates and the interaction between hour and date was consequently not considered. The hourly walking distance was log-transformed before analysis in order to improve normality of residuals.

The average period-wise herbage allowance was determined in order to assess the strength of competition for forage resources which may drive the walking distances in pastures (23). For this, the herbage allowance was regressed on the fixed effect of grazing intensity and the random effect of block. The herbage allowance was square-root transformed before analysis.

To quantify the extent of spatial clustering within period and grazing intensity treatment, each paddock was rasterized into 400 5 × 5 m squares. GPS locations were split into two groups: “active time” included all animal locations during the activity peaks in the morning and afternoon, as determined from the analysis of walking distance per hour. “Other time” included all other animal locations. For each of these sets, the duration (min) spent within each grid cell was calculated. These values were then used to determine the Camargo Index of Evenness across all cells within paddock and period (38) for both groups. The Camargo index allows to assess spatial patterns and the relative distribution of GPS locations within each paddock. Values near zero indicate a patchy distribution and values near one a homogenous distribution (38). This index is, thus, a metric for the requirement of searching to fulfill the herbage intake in relation to the grazing intensity. The Camargo Index was then analyzed in models with the grazing intensity as fixed and block as random effect separately for each period. For other time, the approach was similar.

The relationship between the activity of time spent grazing, (grazing time in min hour^−1^, based on the activity sensor measurements) and the hourly walked distance was analyzed in an analysis of covariance with the walking distance per hour as covariate, the grazing intensity and the interaction of both as fixed and the block as random effect. Variance adjustments were allowed per date in that model. A significance level of *p* ≤ 0.05 was chosen throughout.

All spatial maps were plotted with QGIS (3.10.12 “A Coruña”).

## Results

### Average Daily Walking Distances Within Each Grazing Period

Differences of the daily walking distances between grazing intensities were mostly significant but depended on the grazing period (Tables 3, 4). While in 2017 and autumn of 2019, the daily walking distances were affected by the grazing intensity (Table 3), no effects were found in 2020 and spring of 2019 (although *p* < 0.1). In most periods, walking distances were largest for grazing intensity M (not in 2020), while they were lowest for grazing intensity L in most periods (not in 2017) and those of VL tended to range between them (Table 4). The daily distances varied between 2,592 m (2017 grazing intensity L) and 3,929 m (2020 grazing intensity VL).

**Table 3:** Output of linear mixed effects models for the analyzed parameters of interest during each grazing period.

**Period**	**Variable**	**Fixed and interaction effects**	***F*-value**	***P*-value**
2017	Daily walking distance	Grazing intensity	30.6	*P* < 0.01
	Hourly walking distance	Grazing intensity	0.2	n.s.
		Hour	104.7	*P* < 0.001
		Grazing intensity × hour	3.7	*P* < 0.001
	Herbage on offer	Grazing intensity	10.3	*P* < 0.001
	SD herbage	Grazing intensity	4.8	*P* < 0.1
	Herbage allowance	Grazing intensity	118.8	*P* < 0.001
	Camargo active time	Grazing intensity	7.8	<0.05
	Camargo other time	Grazing intensity	22.5	<0.01
2019 spring	Daily walking distance	Grazing intensity	5.6	*P* < 0.1
	Hourly walking distance	Grazing intensity	1.7	n.s.
		Hour	71.6	*P* < 0.001
		Grazing intensity × hour	5.0	*P* < 0.001
	Herbage on offer	Grazing intensity	75.8	*P* < 0.001
	SD herbage	Grazing intensity	39.2	*P* < 0.01
	Herbage allowance	Grazing intensity	493.7	*P* < 0.001
	Camargo active time	Grazing intensity	5.1	n.s.
	Camargo other time	Grazing intensity	7	n.s.
	Grazing time	Distance	4,064	*P* < 0.001
		Grazing intensity	7.5	*P* < 0.001
		Distance × Grazing intensity	38.7	*P* < 0.001
2019 autumn	Daily walking distance	Grazing intensity	58	*P* < 0.01
	Hourly walking distance	Grazing intensity	1.7	n.s.
		Hour	60.7	*P* < 0.001
		Grazing intensity × hour	2.5	*P* < 0.001
	Herbage on offer	Grazing intensity	74.8	*P* < 0.001
	SD herbage	Grazing intensity	3.4	n.s.
	Herbage allowance	Grazing intensity	8.4	*P* < 0.05
	Camargo active time	Grazing intensity	18.3	<0.05
	Camargo other time	Grazing intensity	17.5	<0.05
2020	Daily walking distance	Grazing intensity	n.s.	n.s.
	Hourly walking distance	Grazing intensity	6.3	n.s.
		Hour	107.1	*P* < 0.001
		Grazing intensity × hour	5.5	*P* < 0.001
	Herbage on offer	Grazing intensity	29.6	*P* < 0.001
	SD herbage	Grazing intensity	11.1	*P* < 0.05
	Herbage allowance	Grazing intensity	15.6	*P* < 0.01
	Camargo active	Grazing intensity	1.7	n.s.
	Camargo other time	Grazing intensity	24.7	<0.05

*Shown are F- and p-values*.

**Table 4:** Estimated means ± se (standard error) of linear mixed effect models for every period.

**Period**	**GI**	**Individual daily distance (m)**	**HO (g DM m^**−**^^**2**^ ± se)**	**SD Herbage (g DM m^**−**^^**2**^ ± se)**	**HA (kg DM LU^**−1**^ ± se)**
2017	M	3, 642 ± 173 b	235 ± 19.1 a	81.3 ± 8.41	455 ± 51.6 a
	L	2, 958 ± 173 a	319 ± 19.1 ab	108.8 ± 8.41	854 ± 70.7 b
	VL	2, 901 ± 173 a	355 ± 19.1 b	119.2 ± 8.41	1, 421 ± 93.4 c
2019 spring	M	3, 542 ± 201	107 ± 7.1 a	56.6 ± 4.31 a	178 ± 14.9 a
	L	2, 592 ± 201	203 ± 7.1 b	89.6 ± 4.31 b	539 ± 25.8 b
	VL	3, 108 ± 142	219 ± 7.1 b	96.4 ± 4.31 b	902 ± 27.9 c
2019 autumn	M	3, 773 ± 92.7 b	99.5 ± 7.64 a	95.2 ± 5.5	265 ± 59.8 a
	L	3, 329 ± 91.4 a	196.8 ± 7.64 b	106.4 ± 5.5	339 ± 67.6 ab
	VL	3, 653 ± 91.4 b	215.1 ± 7.64 b	115.4 ± 5.5	695 ± 96.9 b
2020	M	3, 680 ± 448	80.9 ± 8.96 a	48.9 ± 3.5 a	358 ± 38.4 a
	L	3, 701 ± 402	156.1 ± 8.96 b	63.7 ± 3.5 ab	621 ± 50.7 b
	VL	3, 929 ± 448	172.2 ± 8.96 b	72.0 ± 3.5 b	670 ± 43.0 b

*Lowercase letters: means with different letters are significantly different between GI within year (p < 0.05). GI, grazing intensity; HO, herbage on offer; SD Herbage, standard deviation of herbage on offer; HA, herbage allowance*.

### Average Hourly Walking Distances Within Each Grazing Period

**Figure 1:**
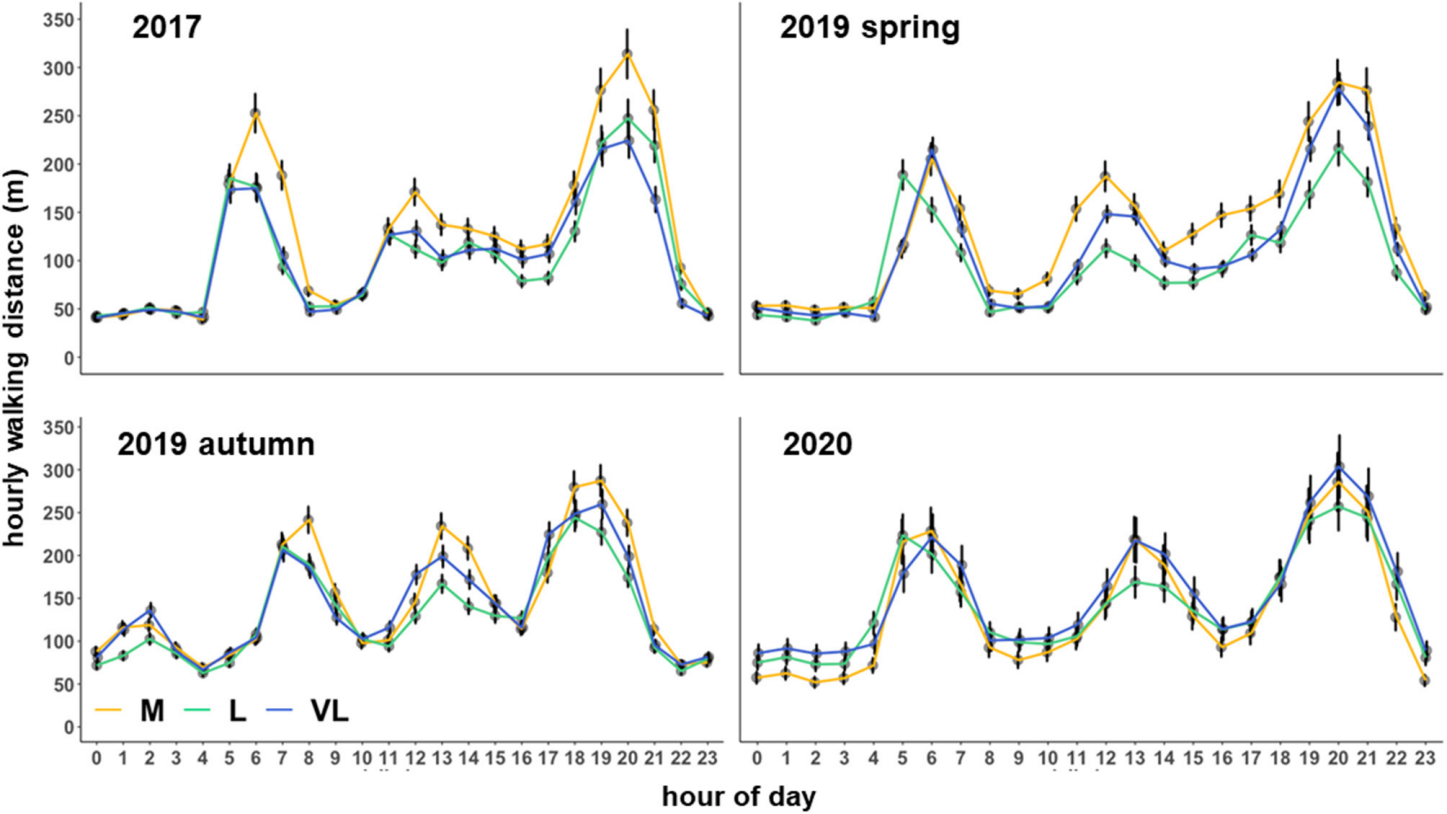
Estimated means (±SE) of the average hourly walking distance (m) as influenced by the grazing period, grazing intensity and hour per day. M, moderate; L, lenient; VL, very lenient grazing intensity.

The interaction between hour per day and the grazing intensity affected the hourly walking distance in all periods (Table 3). A strong diurnal pattern became evident with a shift in the activity peaks during the autumn 2019 period compared with the other periods (Figure 1). The main activity was recorded in the hours 5, 6, 7 a.m. and 7, 8, 9 p.m. (spring and summer periods). In autumn, the activity peaks were narrower, comprising the hours 7, 8 a.m. and 5, 6, 7, 8 p.m. (Figure 1). These time periods were considered as “active time” when the Camargo Index was calculated. On average, they encompassed 40% (M), 39% (L) and 39% (VL) of daily walking distances. The main periods of inactivity occurred during night time and between the activity peaks (Figure 1).

**Figure 2:**
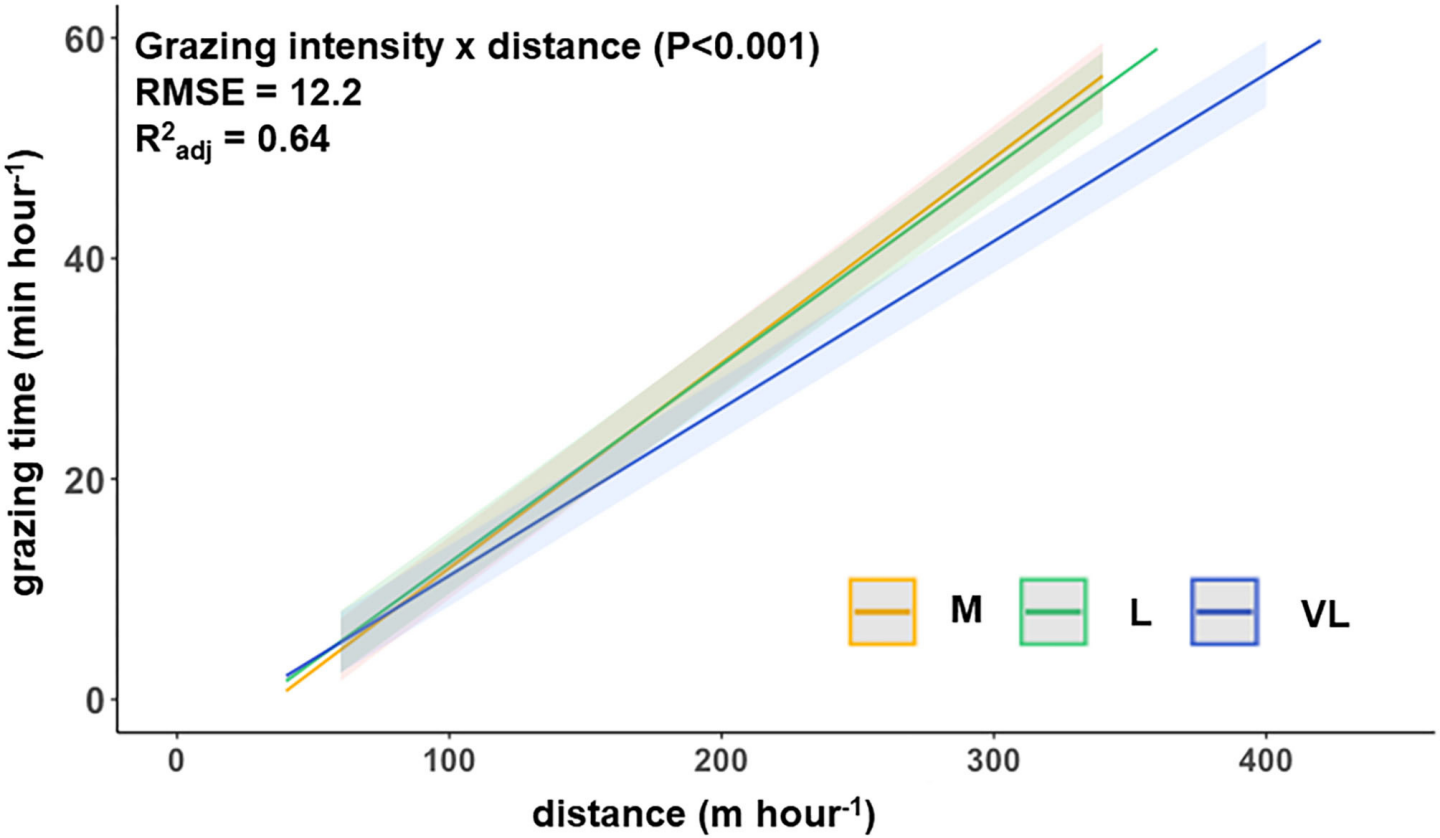
Functional relationship between the hourly walking distance and grazing time per hour (spring 2019) for the three grazing intensities (model prediction). M, moderate; L, lenient; VL, very lenient in spring 2019.

The hourly walking distance and the grazing time (spring 2019) were positively related, with the slope depending on the grazing intensity treatment (Figure 2) as indicated by the significant interaction between distance × grazing intensity.

### Herbage on Offer, Spatial Heterogeneity of Herbage on Offer and Herbage Allowance

The average herbage on offer during each period was affected by the grazing intensity (Tables 3, 4) with a general increase of available herbage from grazing intensity M, over L to VL, but also a visual decline in the available herbage from 2017 until 2020 (Table 4). The values for each measured date are provided in the supplements (Supplementary Figure 1). The herbage allowance was affected by the grazing intensity in all periods (Table 3) and generally increased in the order M < L < VL (Table 4).

There were only significant effects of the grazing intensity on the SD herbage mass in spring of 2019 and 2020 (Table 3) with a clearly lower variability within grazing intensity treatment M compared with L and VL in that period (Table 4). A general trend for increases in SD herbage mass in the rank order M < L ≤ VL, however, became clear for all periods.

### Spatial Distribution in Relation to Grazing Intensity and Period

**Figure 3:**
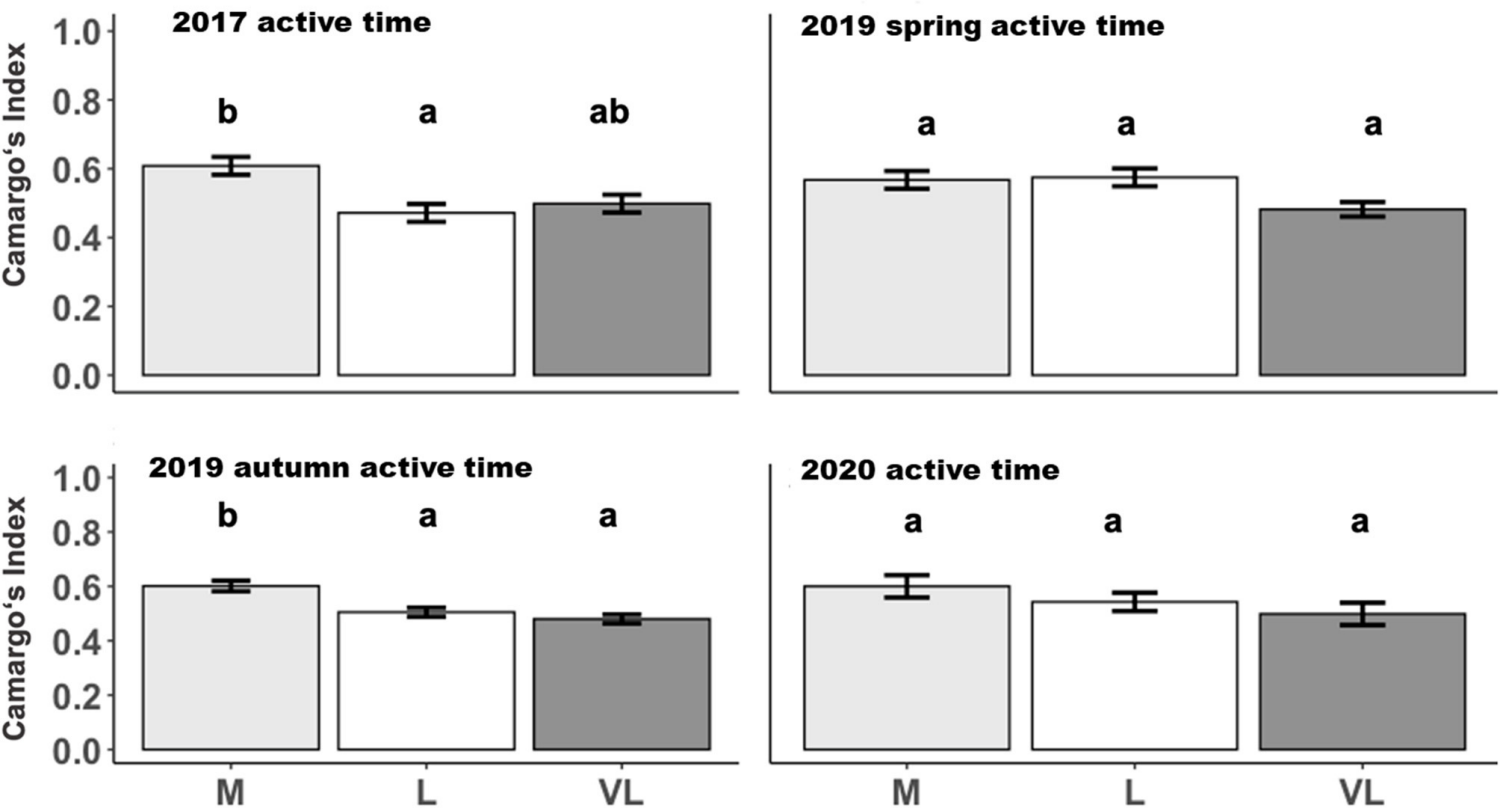
Estimated means (±SE) of the Camargo Index during active time as influenced by the grazing period and grazing intensity. M, moderate; L, lenient; VL, very lenient stocking rate. Identical lowercase letters indicate that means are not different at *p* < 0.05.

**Figure 4:**
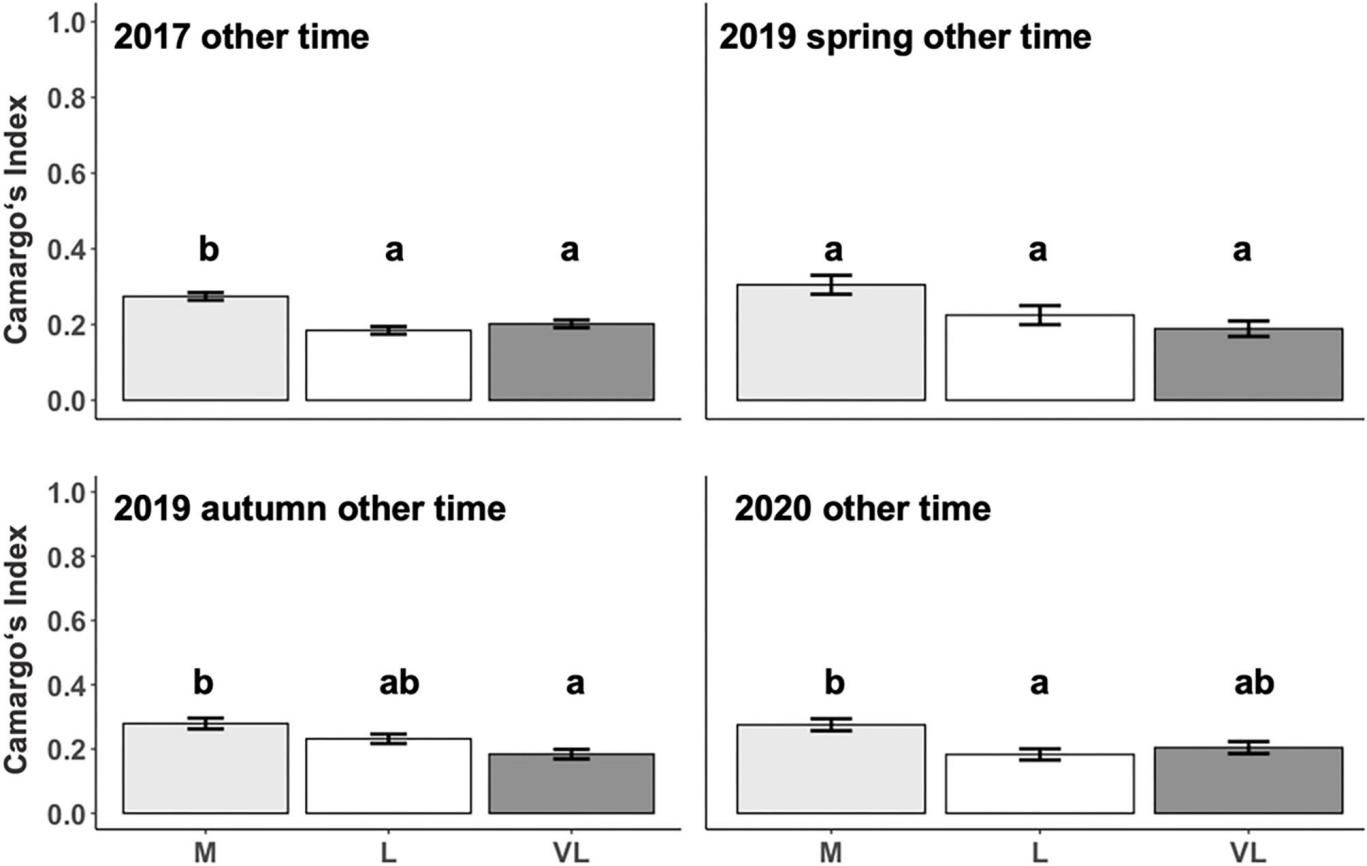
Estimated means (±SE) of the Camargo Index during other time as influenced by the grazing period and grazing intensity. M, moderate; L, lenient; VL, very lenient stocking rate. Identical Lowercase letters indicate that means are not different at *p* < 0.05 within periods.

The Camargo Index was determined for the “active time,” identified as the hours of peak activity according to Figure 1, and for the remaining time (other time) within each period. The Camargo index for the active time was affected by the grazing intensity only in 2017 and 2019 autumn (Table 3), and declined from M to L and VL, indicating a more even distribution within the paddock in grazing intensity M during these periods (Figure 3). This was also confirmed for the Camargo index of the other time periods (Figure 4) which were affected by the grazing intensity in all periods except of spring 2019 (Table 3).

**Figure 5:**
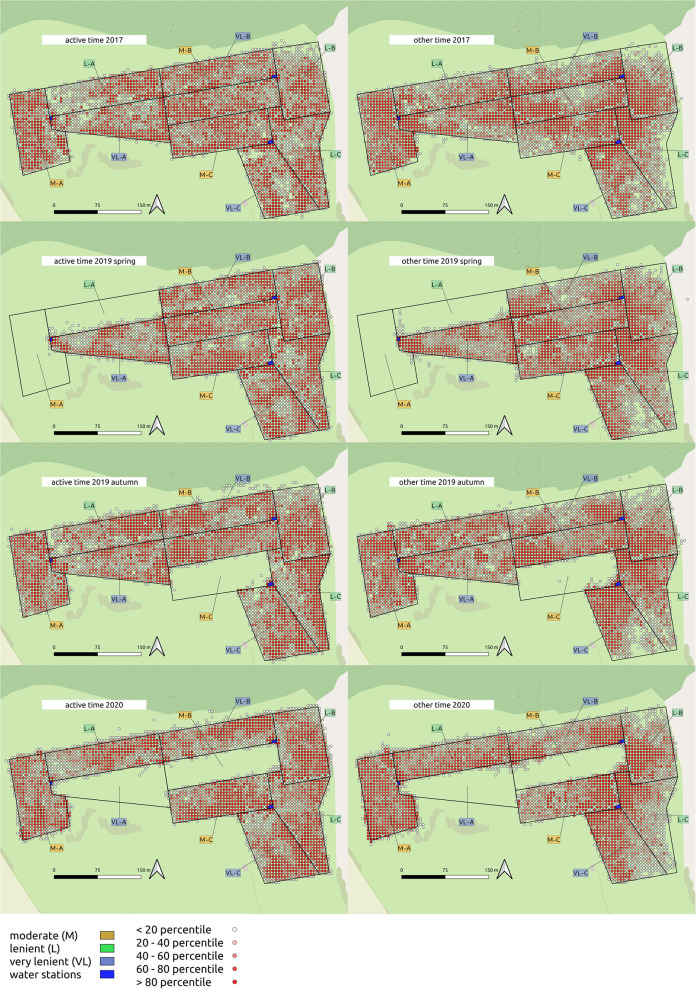
Density maps of cattle location during active time/other time within 5 × 5 m gridcells on the experimental site.

The distribution of spatial points between pastures within each period is given in Figure 5. Time (s d^−1^) spent in each 5 × 5 m grid cell was categorized into five percentiles, visualized as density maps.

## Discussion

While there are many studies on the effects of cattle grazing in different grazing intensities on outcomes for herbage quality (13, 39), biodiversity (3, 40–42), sward botanical composition (43) or productivity (3, 40, 41), the current study is the first to quantify the relationship between cattle movement and grazing intensity, taking into account herbage availability. We hypothesized that (i) cattle activity increased with lower herbage allowance. We further hypothesized that (ii) the spatial distribution of cattle during activity (grazing) peaks is more even under moderate compared to lenient grazing intensity.

### Variation in Herbage Availability and Patterns of Walking Distances in Relation to Grazing Intensity

With an increase in the grazing intensity, the herbage on offer and consequently also the herbage allowance declined (rank order: M < L < VL). However, the spatial heterogeneity of the herbage on offer decreased with increasing grazing intensity (M < VL) which means that the amount of available herbage was lower but more evenly distributed under the moderate grazing treatment M. Increases in the stocking rate and a decline in herbage allowance per individual will cause an increase in the effort of walking on pastures of similar botanical composition (22) – especially under low-input conditions when grassland growth rates are low. Except for the last period, moderate grazing intensity tended to be associated with the greatest effort in walking compared with the other grazing intensities, an effect which became clearly significant in 2017 (Table 4). Hejcmanová et al. (14) investigated behavioral patterns under extensive and intensive continuous grazing and found a clear trend towards longer grazing durations under intensive management. Generally, this larger effort in walking under moderate than under lenient grazing arose from longer durations of the two or three main peak activity phases per 24-h period (Figure 3). However, walking distances were also higher under very lenient than under lenient grazing in some periods (Table 4). Based on the flatter slope between grazing time and walking (Figure 2), this could be attributed to an increased effort in searching of foraging sites.

The mean daily walked distances in the present study ranged between 2,592 and 3,929 m. These values are in accordance with Baker (44), who described a minimum daily activity of 3,000 m on pasture. In a study by Draganova et al. (45), pregnant suckler cows walked between 2,700 and 3,300 m daily on pastures of 8–12 ha in size. Earlier reports state that the daily walking effort of cattle ranges between 2,000 and 6,000 m (22). Consequently, the daily effort in walking is in line with previously reported values (Table 4).

### Spatial Patterns of Movement

In order to differentiate between potential reasons for differences in movement between grazing intensities, we investigated the spatial patterns of movement. As the Camargo index during the active time tended to decrease from M toward VL (Figure 3), we suggest that the larger variability of distribution of the short patch foraging sites is responsible for a stronger clustering in VL. The more even distribution of the animals across the paddocks in M was likely caused by the lower herbage on offer in that treatment and the resulting need to enlarge the grazing area to fulfill the dietetic demand. As described by Perotti et al. (29), in a study on the same experimental site in 2017, the botanical composition differed between short and tall patches. As indicated by Tonn et al. (7), the distribution of the patch classes is mediated by the grazing intensity with larger proportions of short areas under the moderate grazing intensity.

### Heterogeneity/Homogeneity Based on the Standard Deviation of Herbage Mass

It is well established that cattle prefer leafy and digestible vegetation (46) and search actively for it. Cattle are known to develop a spatial memory of the grazing land (47). The pattern of patches seems to be the landmap of the cattle to find preferred forage spots which are repeatedly visited (48). This behavior maximizes the foraging efficiency in terms of forage intake per unit of walking distance (49). However, we found a significantly negative relationship when regressing the walking distance on the standard deviation (SD) of herbage mass as indicator of spatial heterogeneity (*P* < 0.001) (not shown). One has to take into account that the standard deviation of the herbage on offer may be misleading in terms of the actual variability in the spatial distribution of herbage as it is sensitive to the range of values (SD herbage will increase with greater herbage on offer values). Under very lenient grazing, tall avoided areas with large herbage on offer are close to shortly grazed patches with little herbage on offer (7). In contrast to this, under moderate grazing the overall amount of herbage on offer is lower and so is the SD herbage. The very lenient grazing intensity has, thus, a larger amount of unpreferred tall herbage while the moderate treatment has more valuable herbage sources at a lower amount, which both lead to a homogeneous distribution. However, both treatments have the same coefficient of variation (CV) in terms of herbage on offer (not shown). According to Pavlu et al. (31), patches differ in their forage quality and we found a decline of the paddock-scale *in vitro* digestibility from M to VL. When a pasture is stocked with less cattle (as in most cases during our study in VL compared with M) one grazing patch will provide forage resources for a longer duration. Visual cues associated with disparate feed qualities are used by cattle for more efficient forage intake (50), providing evidence for the spatial memory of the grazing livestock. On the contrary, more effort in walking in the moderate grazing treatment is likely a cause of the lower productivity of short patches (10) which requires to enlarge particular grazing areas per individual under higher stocking density in line with Gibb et al. (51). The negative relationship between SD Herbage and walking effort, however, supports our assumption of two different reasons for increased movement. In M, the grazing stations (short patches) provide forage and were evenly distributed but triggered the cattle to enlarge the grazing area during grazing to fulfill the dietetic demand. In VL, the homogeneously distributed tall and mature herbage drove the movement of the cattle to find preferred forage spots.

### Limitations of the Current Study and Variations Among the Periods

In the present study, only one cow per paddock was equipped with a GPS collar which might not fully reflect the potential effect of the group of grazing animals and individual differences on the grazing behavior. Yet, there is indication for the validity of the findings for the following reasons: the experimental setup provides true replication of the grazing intensity treatments at the paddock-level. Among years, the individuals changed between the grazing treatments. In addition, members of a group of animals usually graze simultaneously (52) while only for the resting time and the time spent for ruminating there is a higher variability among different animals within a group (53). However, we suggest that future studies should look into herd dynamics in greater detail to understand effects of the stocking density on the effort for walking.

**Figure 6:**
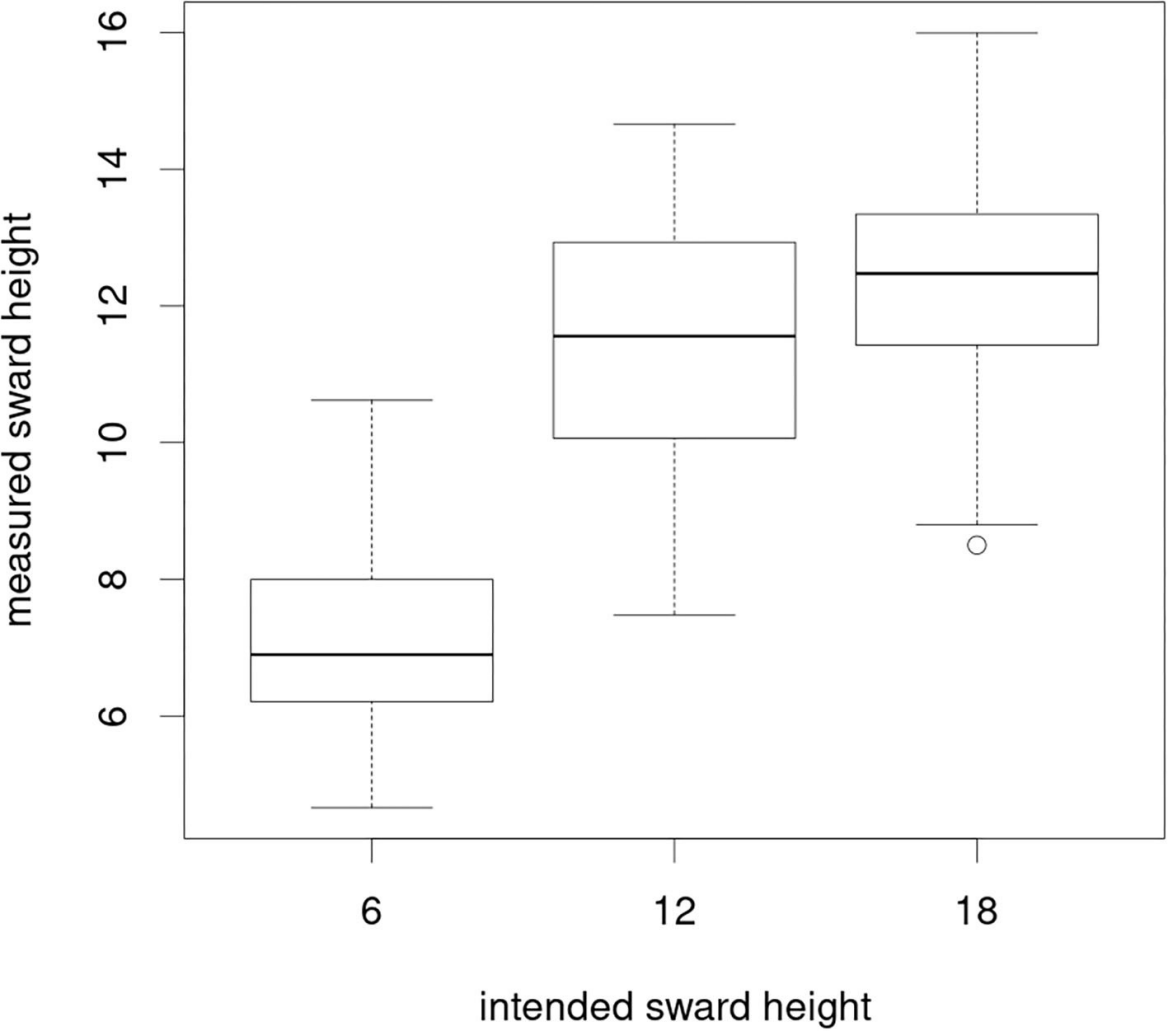
Measured (mean of 50 measurements per paddock taken every 2 weeks) against realized sward heights per grazing intensity across all study periods.

The put-and-take system aims at maintaining sward heights close to the target values by adapting stocking densities to current herbage growth rates, resulting in a gradient of stocking rates across the whole grazing system. The precision with which these aims can be achieved at a given moment strongly depends on the variability of paddock-specific dynamics in grass growth. Sward measurements during the periods showed that the mean measured CSH in grazing intensity M was mainly close to the intended sward height of 6 cm. Measured sward heights under L and VL were close to each other despite different target sward heights (Figure 6). The target sward height of VL of 18 cm was not achieved during our investigation period, which means that the grazing intensities L and VL differ mainly in their herbage allowance but not in the total herbage on offer.

In the periods of 2019 autumn and 2020, the stocking densities between the grazing intensity treatments were nearly the same. Comparable stocking densities result when the actual herbage on offer requires some adjustment in the number of cows stocked per paddock in order to allow for at least 14 days of grazing, which is the rhythm of sward height measurements in the experiment. However, the treatment M is usually stocked earlier in the season so that the annual stocking rates differ clearly between treatments. Nevertheless, it cannot be excluded that the lack of differences in the walking effort between the grazing intensity treatments in 2020 resulted from the fact that the stocking densities among the treatments were the same during that period, even though herbage allowance differed. However, in a study by Dumont et al. (13), the group sizes did not affect the walking distances of individuals. Further research is necessary to prove this point.

Spatial patterns are usually analyzed in larger scale paddocks which give the livestock a higher probability of performing distinct behavioral patterns at specific places (54). Preliminary work had shown that the mean deviation of the GPS signals of the cattle collars used in the present study, were in a range between 0.6 and 1.9 m. As the collars were set to record values every 128 seconds in 2017 and 2019 spring, or every 60 seconds in 2019 autumn and 2020, this noise adds up to the hourly distances of c. 40 m recorded for the nighttime hours.

## Conclusion

Our hypotheses could be confirmed with the present study: (i) cattle activity increased with lower herbage allowance and (ii) the spatial distribution of cattle during active time (grazing peaks) is more even under moderate compared to lenient grazing intensity. However, in our study, cows increased their walking efforts under both the most intensive and also the least intensive grazing treatment. Thus, the herbage availability in terms of herbage allowance and also the spatial distribution (i.e., heterogeneity) of the sward have to be taken into account since all these are drivers for cattle motion. This is relevant information in order to design decision support tools in future precision livestock farming, aiming at a better balance of biodiversity and production targets of grazing systems.

## Data Availability Statement

The datasets generated for this study are available on request to the corresponding author.

## Ethics Statement

The study is in accordance with the German legal and ethical requirements of appropriate animal procedures. The consultation of the Institutional Animal Welfare Body is documented under no. E5/20 by the Animal Welfare Officer of the University of Goettingen.

## Author Contributions

JI, BT, and MK: initiation and supervision of research. DH, MK, and JI: conceptualization. JI and BT: funding acquisition. JH, MK, and BT: data acquisition incl. field measurements. DH, MK, BT, and JH: data analysis. DH (lead), MK, and NG: visualization and writing. NG, MK, JI, and BT: manuscript revision. All authors contributed to the article and approved the submitted version.

## Conflict of Interest

The authors declare that the research was conducted in the absence of any commercial or financial relationships that could be construed as a potential conflict of interest.
