# Supplementary material for: The Effect of Grazing Intensity and Sward Heterogeneity on the Movement Behavior of Suckler Cows on Semi-natural Grassland
Source: Front Vet Sci. 2021 Mar 26;8:639096. doi: 10.3389/fvets.2021.639096 (PMC8032882; doi:10.3389/fvets.2021.639096)
Supplement: Supplementary file 1 [file Data_Sheet_1.PDF]

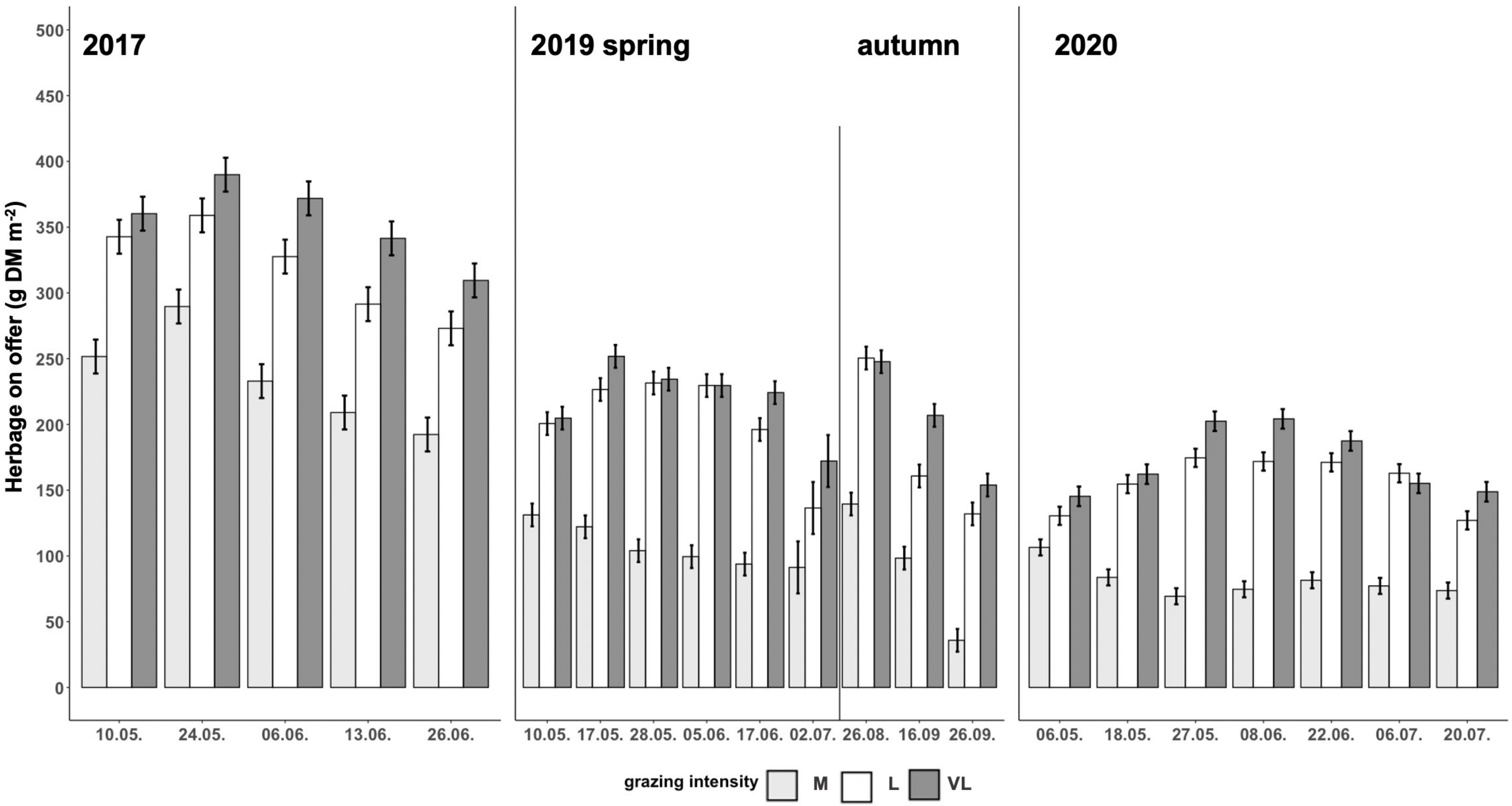

Figure S 1. Mean estimates estimates (±SE) of herbage on offer as determined during each grazing period by regular compressed sward height measurements (50 per date and treatment). Values are calculated from linear-mixed effects models with the grazing intensity and date as well as their interaction as fixed and block and random effects.
